# Prognostic values of normal preoperative serum cancer markers for gastric cancer

**DOI:** 10.18632/oncotarget.11248

**Published:** 2016-08-12

**Authors:** Fan Feng, Li Sun, Zhen Liu, Shushang Liu, Gaozan Zheng, Guanghui Xu, Man Guo, Xiao Lian, Daiming Fan, Hongwei Zhang

**Affiliations:** ^1^ Department of Digestive Surgery, Xijing Hospital, Fourth Military Medical University, 710032, Xi'an, Shaanxi, China

**Keywords:** gastric cancer, CA19-9, AFP, CA125, prognosis

## Abstract

We examined the prognostic value of normal levels of four serum cancer markers, carcinoembryonic antigen (CEA), carbohydrate associated antigen (CA19-9), alpha-fetoprotein (AFP) and cancer antigen 125 (CA125), in gastric cancer patients. Among 1927 gastric cancer patients enrolled in this study, 1477 were male (76.6%) and 450 were female (23.4%). The median age was 57 years (range 20-86). Clinicopathological features and survival times were recorded, and the association between CEA, CA19-9, AFP, and CA125 levels and patient prognosis was analyzed. The optimal cut-off values were 0.71 for CEA (P=0.317), 9.22 for CA19-9 (P=0.009), 3.76 for AFP (P=0.008) and 15.65 for CA125 (P=0.006). Serum CA19-9 levels correlated with gender, age, and tumor depth (all P<0.05); AFP levels correlated with pathological type (P=0.005); and CA125 levels correlated with gender, tumor size, pathological type, tumor depth and lymph node metastasis (all P<0.05). Relatively high levels of CA19-9, AFP and CA125, still within the normal range, were all associated with poor prognosis (5-year overall survival: 70.6% vs 64.2%, P<0.001. 69.6% vs 54.5%, P=0.011. 70.2% vs 54.9%, P<0.001). However, only CA19-9 and AFP levels were independent prognostic predictors. We conclude that the combined assessment of CA19-9, AFP and CA125 levels could have prognostic value in gastric cancer (P<0.001).

## INTRODUCTION

Although the incidence of gastric cancer has decreased worldwide, it is still the fifth most common malignancy and the third leading cause of cancer-related mortality in the world [1]. Surgical resection with extended lymph node clearance remains the only curative therapy for non-metastatic gastric cancer. Even with advances in surgical techniques and adjuvant therapy, the prognosis of advanced gastric cancer is still discouraging due to late diagnosis [2].

A variety of factors are well recognized as prognostic indicators for gastric cancer, including tumor size, tumor depth, lymph node metastasis (LNM), and vessel involvement [3, 4]. In addition, elevated levels of preoperative tumor markers, including CEA [5], CA19-9 [6], AFP [7], and CA125 [8], were demonstrated to be associated with gastric cancer prognosis. However, the prognostic values of these tumor markers within the normal range have not yet been investigated. Therefore, the purpose of this study was to explore the prognostic values of normal, preoperative CEA, CA19-9, AFP, and CA125 levels in gastric cancer.

## RESULTS

**Figure 1:**
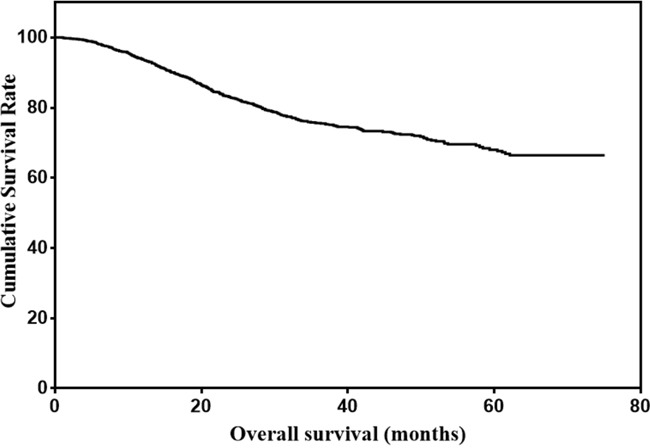
Overall survival of gastric cancer patients with normal serum CEA, CA19-9, AFP, and CA125 levels

**Figure 2:**
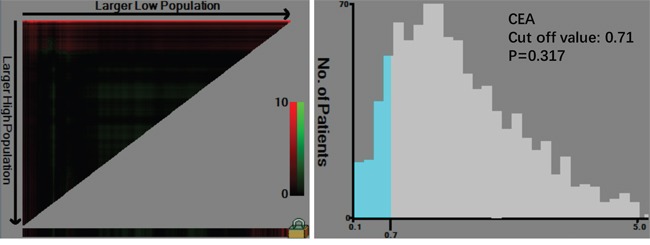
Cut-off value of serum CEA for the prognosis of gastric cancer as calculated using X-tile

**Figure 3:**
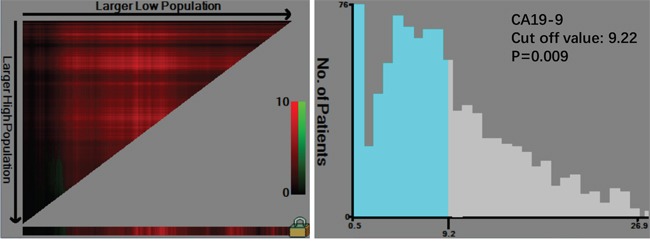
Cut-off value of serum CA19-9 for the prognosis of gastric cancer as calculated using X-tile

**Figure 4:**
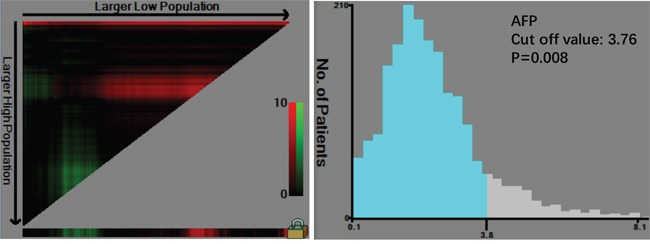
Cut-off value of serum AFP for the prognosis of gastric cancer as calculated using X-tile

**Figure 5:**
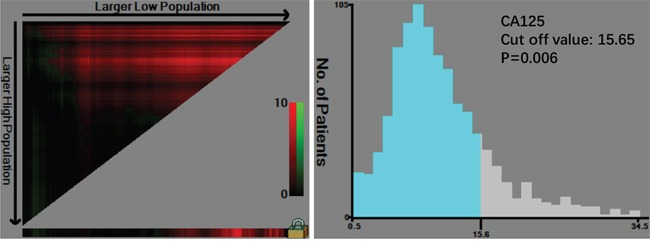
Cut-off value of serum CA125 for the prognosis of gastric cancer as calculated using X-tile

There were 1477 males (76.6%) and 450 females (23.4%) enrolled in this study. The patient age ranged from 20-86 years (median, 57; mean, 56.6). The follow-up time ranged from 1 to 75 months (median, 26.0; mean, 29.7). The 1-, 3- and 5-year overall survival (OS) rates were 93.7%, 75.6% and 68.0%, respectively (Figure 1). The optimal cut-off values of normal serum CEA, CA19-9, AFP, and CA125 for the prognosis of gastric cancer were calculated using X-tile software and are shown in Figures 2–5. The optimal cut-off values were: 0.71 for CEA (P=0.317), 9.22 for CA19-9 (P=0.009), 3.76 for AFP (P=0.008), and 15.65 for CA125 (P=0.006).

**Figure 6:**
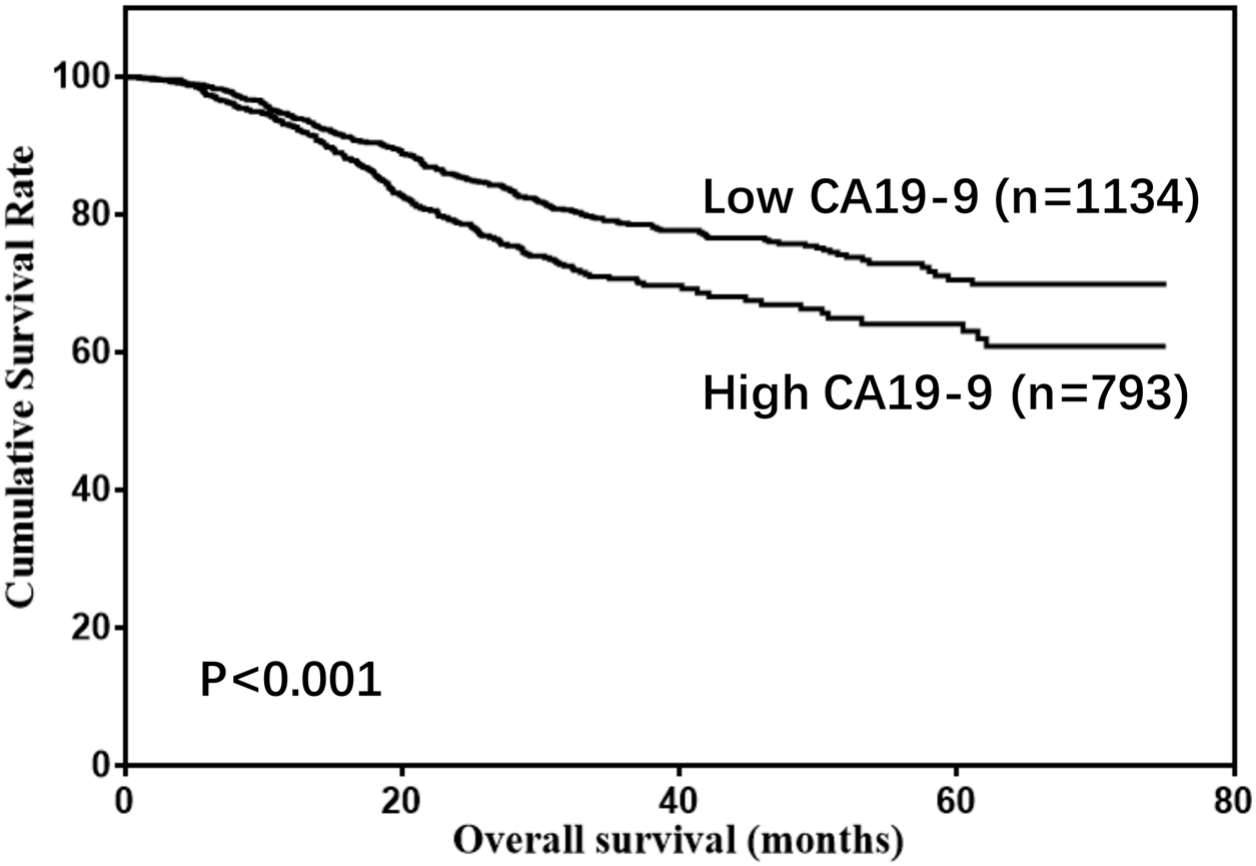
Overall survival of gastric cancer patients according to CA19-9 levels

**Figure 7:**
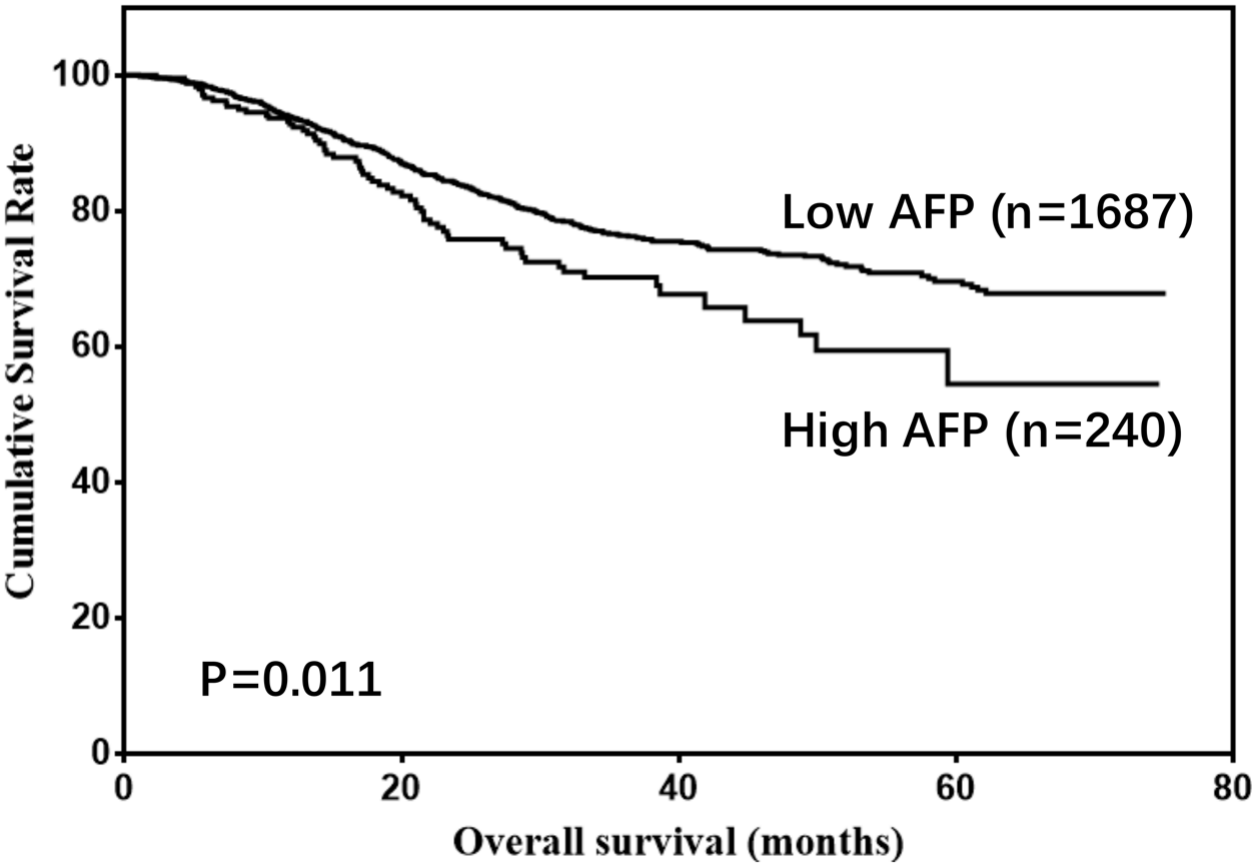
Overall survival of gastric cancer patients according to AFP levels

**Figure 8:**
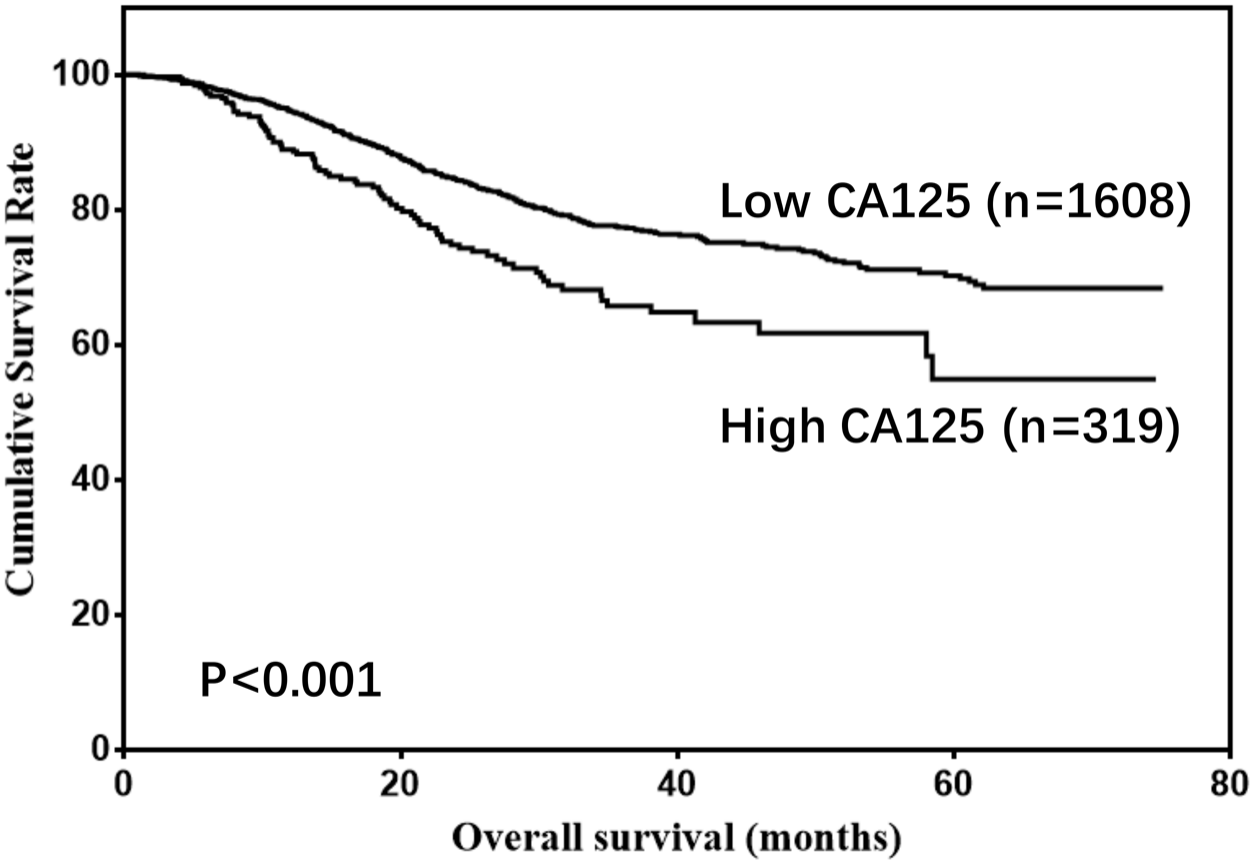
Overall survival of gastric cancer patients according to CA125 levels

Thus, CA19-9, AFP, and CA125 were tested in univariate and multivariate analyses as prognostic predictors of gastric cancer. The univariate analysis showed that age, tumor size, pathological type, tumor depth, LNM, CA19-9, AFP, and CA125 were risk factors for gastric cancer (Table 1). However, only age, tumor size, pathological type, tumor depth, LNM, CA19-9, and AFP were independent prognostic factors according to multivariate analysis (Table 2). Moreover, relatively high levels of CA19-9, AFP, and CA125 were associated with poor prognosis (5-year OS: 70.6% vs 64.2%, P<0.001. 69.6% vs 54.5%, P=0.011. 70.2% vs 54.9%, P<0.001; Figures 6–8). Clinicopathological features between the high- and low-level groups of CA19-9, AFP, and CA125 were analyzed and are summarized in Tables 3–5, respectively. Serum CA19-9 levels were correlated with gender, age, and tumor depth (all P<0.05), serum AFP levels were correlated with pathological type (P=0.005), and serum CA125 levels were correlated with gender, tumor size, pathological type, tumor depth, and LNM (all P<0.05).

**Table 1:** Univariate analysis of risk factors for prognosis of gastric cancer

Prognostic factors	β	Hazard ratio (95% CI)	P value
Gender	0.226	1.253(0.999-1.572)	0.051
Age	0.234	1.264(1.035-1.542)	0.021
Tumor location	−0.037	0.964(0.868-1.070)	0.488
Tumor size	1.291	3.636(2.977-4.441)	0.000
Pathological type	0.554	1.740(1.509-2.007)	0.000
Tumor depth	0.872	2.393(2.124-2.696)	0.000
Lymph node metastasis	0.777	2.174(1.986-2.380)	0.000
CEA	−0.213	0.808(0.606-1.077)	0.808
CA19-9	0.376	1.456(1.194-1.777)	0.000
AFP	0.348	1.417(1.083-1.852)	0.011
CA125	0.493	1.637(1.286-2.085)	0.000

**Table 2:** Multivariate analysis of risk factors for prognosis of gastric cancer

Prognostic factors	β	Hazard ratio (95% CI)	P value
Age	0.247	1.280(1.005-1.630)	0.046
Tumor size	0.623	1.865(1.447-2.405)	0.000
Pathological type	0.262	1.300(1.030-1.640)	0.027
Tumor depth	0.436	1.547(1.293-1.850)	0.000
Lymph node metastasis	0.520	1.683(1.472-1.923)	0.000
CA19-9	0.331	1.393(1.096-1.770)	0.007
AFP	0.459	1.583(1.166-2.149)	0.003

**Table 3:** Comparison of clinicopathological characteristics between two groups

Characteristics	Low CA19-9 (n=1134)	High CA19-9 (n=793)	P value
Gender			0.013
Male	892	585	
Female	242	208	
Age			0.000
≤60	744	425	
>60	390	368	
Tumor location			0.360
Upper third	290	218	
Middle third	218	131	
Lower third	538	389	
Entire	88	55	
Tumor size (cm)			0.369
≤5	858	614	
>5	276	179	
Pathological type			0.890
Well differentiated	145	100	
Moderately differentiated	276	187	
Poorly differentiated	670	480	
Signet ring cell or Mucinous	43	26	
Tumor depth			0.004
T1	307	201	
T2	192	134	
T3	384	228	
T4	251	230	
Lymph node metastasis			0.052
N0	527	319	
N1	199	148	
N2	170	134	
N3	238	192	

**Table 4:** Comparison of clinicopathological characteristics between two groups

Characteristics	Low AFP (n=1687)	High AFP (n=240)	P value
Gender			0.105
Male	1303	174	
Female	384	66	
Age			0.235
≤60	1015	154	
>60	672	86	
Tumor location			0.746
Upper third	444	64	
Middle third	300	49	
Lower third	818	109	
Entire	125	18	
Tumor size (cm)			0.304
≤5	1295	177	
>5	392	63	
Pathological type			0.005
Well differentiated	209	36	
Moderately differentiated	426	37	
Poorly differentiated	989	161	
Signet ring cell or Mucinous	63	6	
Tumor depth			0.185
T1	439	69	
T2	294	32	
T3	542	70	
T4	412	69	
Lymph node metastasis			0.949
N0	744	102	
N1	302	45	
N2	267	37	
N3	374	56	

**Table 5:** Comparison of clinicopathological characteristics between two groups

Characteristics	Low CA125 (n=1608)	High CA125 (n=319)	P value
Gender			0.000
Male	1268	209	
Female	340	110	
Age			0.345
≤60	983	186	
>60	625	133	
Tumor location			0.334
Upper third	429	79	
Middle third	295	54	
Lower third	772	155	
Entire	112	31	
Tumor size (cm)			0.000
≤5	1255	217	
>5	353	102	
Pathological type			0.038
Well differentiated	217	28	
Moderately differentiated	392	71	
Poorly differentiated	939	211	
Signet ring cell or Mucinous	60	9	
Tumor depth			0.003
T1	436	72	
T2	287	39	
T3	504	108	
T4	381	100	
Lymph node metastasis			0.000
N0	739	107	
N1	286	61	
N2	252	52	
N3	331	99	

**Figure 9:**
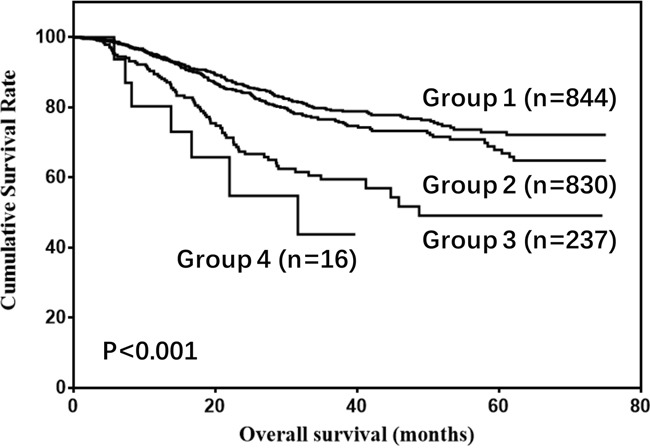
Overall survival of gastric cancer patients according to the combination of CA19-9, AFP, and CA125 levels

The prognostic value of the combination of CA19-9, AFP, and CA125 for gastric cancer was also evaluated. Gastric cancer patients were divided into four groups: Group 1) low CA19-9, low AFP and low CA125; Group 2) high CA19-9, low AFP, and low CA125; or low CA19-9, high AFP, and low CA125, or low CA19-9, low AFP, and high CA125; Group 3) high CA19-9, high AFP, and low CA125, or high CA19-9, low AFP, and high CA125; or low CA19-9, high AFP, and high CA125; and Group 4) high CA19-9, high AFP, and high CA125. As shown in Figure 9, OS rates were gradually reduced along with increasing levels of CA19-9, AFP, and CA125 (P<0.001).

## DISCUSSION

A large number of studies have investigated the predictive value of elevated preoperative serum CEA, CA19-9, AFP, and CA125 levels for the prognosis of gastric cancer. However, no studies have investigated the prognostic value of normal CEA, CA19-9, AFP, and CA125 levels in gastric cancer patients. The present study found that relatively high levels of CA19-9, AFP, and CA125 within the normal limits were associated with a poor prognosis in gastric cancer.

Elevated preoperative serum CEA, CA19-9, AFP, and CA125 have been previously associated with a poor prognosis in gastric cancer. A meta-analysis of 14,651 gastric cancer patients found that elevated serum CEA was an independent prognostic risk factor [5]. Similarly, another meta-analysis of 11,408 gastric cancer patients showed that elevated serum CA19-9 was associated with a poor prognosis [9]. Elevated serum AFP has also been associated with a poor prognosis in gastric cancer, and it can predict liver metastasis after radical resection [7, 10, 11]. Elevated serum CA125 levels have been associated with peritoneal metastasis of gastric cancer [12, 13], and elevated CA125 in peritoneal lavage was associated with peritoneal dissemination and a poor prognosis [14].

Although gastric cancer patients with normal, preoperative serum CEA, CA19-9, AFP, and CA125 had favorable prognoses, the prognostic value of relatively high levels of the four tumor markers within the normal limits was important to test. We found that relatively high levels of CA19-9, AFP, and CA125 were associated with poor prognosis of gastric cancer. However, serum CEA did not have prognostic value. Further, the combination of relatively high levels of CA19-9, AFP, and CA125 increased the prognostic value for gastric cancer, even though the levels were all within the normal limits. These results provide new insights into the prognosis of gastric cancer patients with normal, preoperative tumor markers.

Levels of CEA, CA19-9, AFP, and CA125 are also widely used to monitor recurrence or metastasis of gastric cancer after radical gastrectomy. Patients with normal postoperative CEA levels have a better prognosis [15]. Similarly, Kwon et al. reported that postoperative normalization of CA19-9 can be a surrogate for potentially curative surgical treatment and can be used as a prognostic factor for gastric cancer [16]. However, the predictive value of these tumor markers within normal limits after radical gastrectomy still needs further investigation.

A strong correlation between tumor marker levels and clinicopathological characteristics has been reported previously. Serum CEA levels were associated with tumor depth, LNM, TNM stage, and liver metastasis [17, 18]. Serum CA19-9 levels were also associated with tumor depth and LNM, as well as lymphatic-vascular invasion [19, 20]. Serum AFP levels were associated with LNM, vascular invasion, and liver metastasis [21], and serum CA125 was correlated with vascular invasion, LNM, and tumor stage [22]. We found that serum CA19-9 levels were correlated with gender, age, and tumor depth, serum AFP levels were correlated with pathological type, and serum CA125 levels were correlated with gender, tumor size, pathological type, tumor depth, and LNM.

There are several limitations to our study. First, it was a retrospective study of a single center's experience. Multi-center studies are needed to verify these findings. Second, the sample size was not large, especially for the patients with high levels of CA19-9, AFP, and CA125, which may result in bias during analysis. Third, the prognostic value of normal, CEA, CA19-9, AFP, and CA125 levels for gastric cancer patients after radical gastrectomy during follow-up were not investigated. Nonetheless, we conclude that relatively high levels of preoperative serum CA19-9, AFP, and CA125 within the normal limits are associated with poor prognosis of gastric cancer. Thus, the combination of CA19-9, AFP and CA125 levels could further increase the predictive value for the prognosis of gastric cancer.

## MATERIALS AND METHODS

This study was performed in the Xijing Hospital of Digestive Diseases affiliated with the Fourth Military Medical University. From September 2008 to March 2015, a total of 1927 gastric cancer patients were enrolled. The inclusion criteria were as follows: 1) no neoadjuvant chemotherapy, 2) radical D2 gastrectomy, 3) normal, preoperative serum CEA, CA19-9, AFP, and CA125 levels, and 4) with follow-up data. This study was approved by the Ethics Committee of Xijing Hospital, and written informed consent was obtained from all patients before surgery.

The four serum tumor markers were detected within 7 days before surgery. The cut-off values for serum CEA, CA19-9, AFP, and CA125 were 5 ng/ml, 27 U/ml, 8.1 ng/ml, 35 U/ml. Preoperative data including gender, age, tumor location, serum CEA, serum CA19-9, serum AFP and serum CA125 were recorded. All patients were treated with proximal, distal, or total gastrectomy with D2 lymphadenectomy. The surgical procedure was based on the recommendations of the Japanese Gastric Cancer Treatment Guidelines [23]. The depth of primary tumor and degree of lymph node involvement were defined according to the TNM classification. Tumor size, differentiation status, tumor depth, and LNM data were also collected during the pathological examination. The patients remained in follow-up until November, 2015 with enhanced chest and abdominal CT and gastroscopy every 3 months.

Data were processed using SPSS 22.0 for Windows (SPSS Inc., Chicago, IL, USA). The optimal cut-off values of serum CEA, CA19-9, AFP, and CA125 for prognosis of gastric cancer were calculated using X-tile software [24]. Discrete variables were analyzed using Chi-square or Fisher's exact tests. Significant risk factors identified by univariate analysis were further assessed by multivariate analysis using logistic regression. OS was analyzed by Kaplan-Meier method. A P value of 0.05 was considered statistically significant.
